# Diagnosis and Treatment of Liver Disease: Current Trends and Future Directions

**DOI:** 10.7759/cureus.49920

**Published:** 2023-12-04

**Authors:** Hina Wazir, Marium Abid, Binish Essani, Hira Saeed, Muhammad Ahmad Khan, FNU Nasrullah, Usama Qadeer, Ayesha Khalid, Giustino Varrassi, Muhammad Ali Muzammil, Areeba Maryam, Abdul Rehman Shah Syed, Abdul Ahad Shah, Satish Kinger, Farhan Ullah

**Affiliations:** 1 Internal Medicine, Khyber Medical College, Peshawar, PAK; 2 Medicine, Jinnah Medical and Dental College, Karachi, PAK; 3 Medicine, Federal Medical College, Islamabad, PAK; 4 Medicine, Medical University of Sofia, Sofia, BGR; 5 Internal Medicine, Shadab Medical Center, Karachi, PAK; 6 Medicine, Allama Iqbal Medical College, Lahore, PAK; 7 Medicine, Fatima Memorial Hospital College of Medicine and Dentistry, Lahore, PAK; 8 Pain Medicine, Paolo Procacci Foundation, Rome, ITA; 9 Internal Medicine, Dow University of Health Sciences, Karachi, PAK; 10 Emergency Medicine, Holy Family Hospital, Rawalpindi, PAK; 11 Medicine, Dow University of Health Sciences, Karachi, PAK; 12 Dermatology, Medical College, Dow University of Health Sciences, Karachi, PAK; 13 Medicine, Civil Hospital Karachi, Karachi, PAK; 14 Internal Medicine, Khyber Teaching Hospital, Peshawar, PAK

**Keywords:** precision medicine in liver disease, future directions, contemporary approaches, treatment strategies, diagnosis trends, liver disease

## Abstract

This narrative review delves into the intricate landscape of liver diseases, providing a comprehensive background of the diverse conditions that afflict this vital organ. Liver diseases, ranging from viral hepatitis and non-alcoholic fatty liver disease (NAFLD) to cirrhosis and hepatocellular carcinoma (HCC), pose significant global health challenges. Understanding these diseases' multifaceted origins and progression is pivotal for developing effective diagnostic and therapeutic strategies. The epidemiology and etiology of liver diseases emphasize the global impact of viral hepatitis, with hepatitis B and C as significant contributors. Concurrently, the rising prevalence of NAFLD, linked to lifestyle factors and metabolic syndrome, underscores the intricate relationship between modern living and liver health. Chronic liver diseases often evolve insidiously, progressing from inflammation to fibrosis and, ultimately, to cirrhosis - a stage characterized by irreversible scarring and compromised function. The heightened risk of HCC in advanced liver disease stages further underscores the urgency of effective diagnostic and therapeutic interventions. The evolving landscape of non-invasive diagnostic tools is explored for their role in enabling early detection and accurate staging of liver diseases. In the realm of treatment, there is a continuous transition toward personalized medicine, customized to suit the unique profiles of individual patients. This shift encompasses a broad spectrum, ranging from personalized pharmacological interventions to lifestyle modifications and surgical options. Delving into innovative therapies, such as gene editing and immunomodulation, offers a glimpse into the promising future directions that have the potential to redefine the landscape of liver disease diagnosis and treatment.

## Introduction and background

In the intricate tapestry of human health, the liver emerges as a silent guardian, diligently performing myriad functions crucial to our well-being. However, this unsung hero is increasingly facing formidable challenges in the form of liver diseases - a diverse array of disorders that cast a shadow over its vital roles. Our exploration into the world of liver health unveils the complexity of these diseases and the urgency to understand, diagnose, and treat them effectively. The canvas of liver diseases spans continents, affecting diverse populations and communities. To grasp the magnitude of this global challenge, let us first focus on the viral instigators - hepatitis B and C. According to the World Health Organization (WHO), these viral foes collectively afflict more than 325 million individuals worldwide [1]. The silent nature of these infections often conceals their prevalence, making them a substantial contributor to the global burden of liver diseases. Yet, the narrative does not end here. Another protagonist in the story of liver health is non-alcoholic fatty liver disease (NAFLD). This condition, intricately linked to the modern lifestyle characterized by sedentary habits and diets laden with sugars and fats, has stealthily become a pervasive concern. Research indicates that approximately 25% of the global population is grappling with NAFLD [2]. It is a statistic that transcends mere numbers, representing the lived experiences of individuals across diverse backgrounds. The repercussions of liver diseases extend beyond numerical prevalence and permeate the fabric of individual lives and societal structures.

The liver, a multi-functional organ involved in detoxification and nutrient metabolism processes, is integral to our overall health. When diseases compromise their functions, the consequences are profound. Consider the journey of an individual grappling with a liver ailment. Symptoms might be subtle or absent in the early stages, allowing the disease to progress silently. As it advances, however, the impact becomes tangible - fatigue, jaundice, abdominal pain, and a cascade of other symptoms surface. The effects reverberate within the individual, families, and communities [3]. From a broader perspective, the economic implications are noteworthy. The costs associated with liver diseases not only encompass medical expenses but also extend to diminished productivity due to illness and the long-term burden of chronic care. It is a complex interplay between health and economics, and effective intervention at the right time becomes crucial for mitigating both individual suffering and societal costs [4]. This brings us to the crux of our exploration - the significance of effective diagnosis and treatment in liver diseases. The elusive nature of these conditions, often presenting with mild or no symptoms in their early stages, underscores the critical need for timely detection. Early diagnosis becomes a linchpin for successful management and improved outcomes [4]. The urgency is accentuated by the fact that liver diseases, if left unchecked, tend to progress insidiously. Chronic hepatitis can evolve into cirrhosis - a stage marked by irreversible scarring of the liver tissue. This, in turn, heightens the risk of hepatocellular carcinoma (HCC), a formidable type of liver cancer. The trajectory from silent progression to critical stages is a stark reminder of the importance of vigilance and proactive healthcare measures. The impact of liver diseases on global health is not a static narrative; it is a dynamic saga with evolving chapters. As we delve into this complex landscape, it becomes apparent that our understanding of liver diseases is expanding, and our approaches to diagnosis and treatment must evolve [5]. In the following pages, our journey will navigate the current trends and future directions in liver disease diagnosis and treatment.

This exploration is not confined to the realms of laboratories and medical offices; it is a journey that intertwines science with humanity, seeking clinical advancements and compassionate solutions. We will traverse the advancements in diagnostic modalities, from the intricacies of advanced imaging techniques to the nuances of serological biomarkers and molecular diagnostics. The evolving landscape of non-invasive diagnostic tools will unfold, showcasing their role in early detection and accurate staging of liver diseases. Simultaneously, we will embark on a quest through the therapeutic landscapes, exploring the pharmacological interventions, lifestyle modifications, and surgical options that form the arsenal against liver diseases. The shift towards personalized medicine, tailoring treatments to individual profiles, is poised to emerge as a beacon of hope, recognizing and addressing the inherent heterogeneity within liver diseases. Our narrative will extend into the realms of emerging therapies, from the groundbreaking potential of gene editing to the promises held by immunomodulation. As we peer into the future, we will unravel ongoing research endeavors and clinical trials poised to reshape the landscape of liver disease management. The journey does not conclude with diagnostics and treatments alone; it extends into digital health and telemedicine. Technology integration is pivotal in enhancing accessibility, monitoring liver diseases, and optimizing long-term care for individuals grappling with liver diseases. This narrative is not just an exposition of facts and figures; it is a call to action for collaborative progress in understanding and managing liver diseases. It invites clinicians, researchers, policymakers, and the broader community to unite in pursuing innovative solutions and compassionate care. We transcend the boundaries of disciplines and specialties as we traverse the terrain of liver disease diagnosis and treatment. We recognize that each statistic represents a life, each advancement in diagnostics brings hope, and each step toward personalized treatment is a stride toward improving the human experience. In the following review, let us delve deeper into the intricate world of liver diseases, not merely as a medical challenge but as a shared endeavor to enhance lives and build a healthier global community.

## Review

Methodology

An extensive literature search was performed to find pertinent research, papers, and reviews related to the diagnosis and treatment of liver disease. The electronic databases PubMed, Scopus, and Web of Science were searched using a set of keywords, including "liver disease," "diagnosis," "treatment," "current trends," and "future directions." The synthesized findings underwent a rigorous critical evaluation and were then presented narratively. The focus was on summarizing the present condition of liver disease diagnosis and therapy, with particular attention given to significant advancements, obstacles, and areas of little understanding. The correlations across various research and trends' progression over time were analyzed. Throughout the assessment process, ethical questions were thoroughly considered. The study ensured proper citation and credit of the original authors. Ethical approval was unnecessary as the study involved analyzing preexisting published data.

Epidemiology of liver diseases

The liver, an essential organ in the human body, performs pivotal functions in metabolism, detoxification, and digestion. Regrettably, it is also vulnerable to many ailments, significantly affecting countless lives globally. This essay explores the epidemiology of two prominent liver illnesses, namely viral hepatitis (particularly hepatitis B and C) and NAFLD. We will examine the worldwide impact, geographical spread, and increasing occurrence of these illnesses, providing insight into their connection with lifestyle variables and metabolic syndrome.

Viral Hepatitis: A Global Challenge

Viral hepatitis, which includes hepatitis B and C, poses a substantial worldwide health problem. These infections, primarily spread through tainted blood and other bodily fluids, impact millions of people, resulting in both acute and chronic liver disorders, such as cirrhosis and HCC. Hepatitis B poses a significant public health issue, affecting approximately 292 million individuals worldwide who are living with chronic infections [1]. Although vaccines have successfully decreased disease occurrence in various areas, there are still difficulties in achieving widespread vaccination, especially in low-income nations [5]. Asia and sub-Saharan Africa bear a disproportionate cost. The intricate interaction between socioeconomic factors, healthcare infrastructure, and vaccination strategies influences the epidemiology of hepatitis B. It is crucial to continue improving vaccination rates, especially among groups and places that are at high risk or have limited resources, to reduce the prevalence of this long-lasting viral infection. Hepatitis C is commonly known as the "silent epidemic" since it typically does not cause noticeable symptoms in the early stages. Approximately 71 million individuals globally are believed to be afflicted with chronic hepatitis C infection [1]. The burden distribution is disproportionately skewed, with a significant frequency in specific regions, namely Eastern Europe, Central Asia, and North Africa. The introduction of direct-acting antivirals (DAAs) has significantly transformed the treatment options for hepatitis C, providing excellent success rates in curing the disease and reducing the length of treatment [6]. Nevertheless, obstacles concerning the availability of diagnosis and treatment remain, particularly in settings with limited resources. Tackling these problems is essential for attaining worldwide objectives of hepatitis C eradication.

Non-Alcoholic Fatty Liver Disease: The Silent Epidemic Within

Over the past several years, there has been a notable change in the field of liver illnesses, as NAFLD has emerged as a prominent source of long-term liver issues. NAFLD is distinguished by the buildup of lipids in the liver of individuals who do not have a notable history of alcohol intake. It encompasses a range of diseases, spanning from basic steatosis to non-alcoholic steatohepatitis (NASH), fibrosis, and cirrhosis. The increase in NAFLD is closely connected to contemporary lifestyle issues, such as lack of physical activity, bad eating habits, and the growing occurrence of metabolic syndrome. The worldwide increase in obesity rates has played a crucial role in the escalating epidemic of NAFLD [7]. Insulin resistance, a vital element of metabolic syndrome, is a primary factor in the development of NAFLD, resulting in fat buildup in the liver. The complex correlation between NAFLD and metabolic syndrome highlights the significance of implementing a comprehensive strategy for its prevention and treatment. Regular physical activity and nutritional treatments are essential components in managing NAFLD. Focusing on these changeable risk factors enhances liver well-being and tackles broader public health issues linked to obesity and metabolic syndrome. The worldwide incidence of NAFLD is increasing, with estimates indicating that almost 25% of the global population is impacted [7]. The illness has transcended its previous limitation to wealthy nations and has now become a worldwide issue, affecting individuals from many socioeconomic backgrounds. The increasing frequency of NAFLD has significant ramifications, placing a considerable strain on healthcare systems globally. NAFLD is closely associated with an elevated susceptibility to cardiovascular illnesses, type 2 diabetes, and other metabolic disorders [8]. The societal and economic effect is significantly increased as the disease progresses to advanced stages, such as NASH and cirrhosis. To tackle the increasing occurrence of NAFLD, it is necessary to adopt a comprehensive approach that includes public health policies, early identification, and specific therapies. Public awareness campaigns and policy actions promoting healthy lives are crucial elements in curbing the spread of this unnoticed epidemic.

Etiology and risk factors

The liver, the body's powerhouse, is an extraordinary organ with diverse tasks. Regrettably, the liver is prone to a variety of diseases, and comprehending the elements that contribute to liver disorders is essential for preventive and efficient treatment. This essay explores the causes and circumstances that increase the likelihood of developing liver illnesses, with a particular emphasis on alcoholic liver disease (ALD). We will examine the significant influence of alcohol use on liver health and elucidate the complex pathways that contribute to ALD. Before exploring the intricacies of liver illnesses, it is crucial to acknowledge the liver's importance in preserving our general state of health. The liver is a central hub for metabolism, performing essential tasks in digestion, detoxification, and nutrition storage. Although the liver is highly resistant, it is susceptible to numerous disorders, and comprehending their origins is crucial. Liver diseases comprise a range of ailments, including viral infections, metabolic disorders, and autoimmune conditions. To understand the causes of these conditions, we need to consider internal and external variables that contribute to their formation. *Viral Hepatitis: Silent Invaders*

Hepatitis viruses (A, B, C, D, and E) are responsible for viral hepatitis, which poses a significant health burden worldwide. These viruses infiltrate the liver, causing inflammation and perhaps long-term harm. Hepatitis B and C are enduring dangers, primarily transmitted through infected blood, unprotected sexual intercourse, and transmission from mother to child during childbirth [9].

Non-Alcoholic Fatty Liver Disease: The Modern Epidemic

The contemporary pandemic NAFLD, commonly linked to obesity and metabolic syndrome, has become a widespread liver disorder. It entails the buildup of fat in the liver cells, which may advance to NASH and fibrosis. NAFLD development is influenced by insulin resistance, dietary behaviors, and hereditary factors [7].

Alcoholic Liver Disease: When Cheers Turn to Tears

ALD is closely associated with the use of alcohol. Prolonged and excessive consumption of alcohol can lead to a range of liver conditions, such as fatty liver, alcoholic hepatitis, and cirrhosis. The risk of ALD is determined by factors such as the amount and duration of alcohol consumption, genetic predisposition, and the presence of concurrent liver diseases [10]. Alcohol, a commonly used substance to facilitate social interactions, has a two-fold effect on the liver. While moderate consumption is generally considered appropriate, excessive and extended intake can disrupt the balance and lead to a series of harmful effects on the liver. Exercising moderation is crucial regarding alcohol use. According to the National Institute on Alcohol Abuse and Alcoholism (NIAAA), moderate drinking is defined as consuming no more than one alcoholic beverage per day for women and no more than two alcoholic beverages per day for men [10]. Within these boundaries, the liver may frequently manage without substantial damage. Prolonged and excessive alcohol use surpasses the liver's capacity to metabolize and cleanse. The liver metabolizes alcohol using enzymes such as dehydrogenase and cytochrome P450 2E1. However, excessive alcohol use beyond the liver's degree can lead to the development of liver disorders [11]. Several factors, such as the amount and frequency of alcohol consumption, individual tolerance, hereditary predisposition, and the presence of other medical disorders, impact alcohol-related liver damage. Specific populations, such as persons with a past of excessive alcohol consumption or those with pre-existing liver conditions, have a higher vulnerability to ALD [11].

Mechanisms Leading to Alcoholic Liver Disease: Unraveling the Molecular Tapestry

To comprehend the transition of alcohol from a substance that facilitates social interactions to a harmful agent for the liver, one must explore the complex molecular and cellular processes occurring within the liver. The process commences with the liver metabolizing alcohol. Alcohol dehydrogenase and cytochrome P450 2E1 are enzymes that metabolize alcohol, transforming it into acetaldehyde, which is a harmful compound. Acetaldehyde is subsequently metabolized into acetate, which can be utilized by the organism as a source of energy [11]. Chronic and excessive alcohol intake results in oxidative damage in the liver. Reactive oxygen species, produced during the process of alcohol metabolism, cause harm to cells, leading to inflammation and ultimately resulting in liver damage [12]. Inflammation is the liver's reaction to injury, typically as a defensive mechanism. Nevertheless, long-term alcohol consumption maintains a state of inflammation, leading to a continuous activation of the immune system that contributes to liver damage and the advancement of ALD [12]. Hepatic stellate cells, typically quiescent, undergo activation in response to liver damage. This process triggers the accumulation of extracellular matrix proteins, leading to the development of liver fibrosis. As fibrosis progresses, it disrupts the structure of the liver, leading to a decline in its normal functioning [12]. In some instances, this process ultimately results in cirrhosis, which is a severe form of liver scarring that occurs in the later stages. Cirrhosis denotes an irreversible impairment that significantly augments the likelihood of consequences, such as liver failure and HCC [13].

Progression of liver diseases

The progression of liver diseases is a complex journey, marked by various stages that profoundly impact liver function. This essay explores the stages of liver disease progression, from inflammation to cirrhosis, emphasizing the consequential risk of HCC and strategies for early detection. Liver diseases evolve through distinct stages, imprinting each phase on the organ's structure and function. Understanding this progression is essential for effective management and timely interventions. The journey often begins with inflammation, the liver's response to injury. Various insults, including viral infections, excessive alcohol consumption, or metabolic conditions, can trigger inflammation. At this stage, the liver may still exhibit remarkable resilience, and inflammation can be managed and even reversed [13]. If inflammation persists, it can lead to the deposition of scar tissue, a process known as fibrosis. Fibrosis is a silent yet crucial stage where the liver's architecture begins to change, impairing its normal function. Early detection and intervention during this phase can prevent further progression [14]. Unchecked inflammation and fibrosis may culminate in cirrhosis - a late-stage scarring of the liver. Cirrhosis represents irreversible damage, significantly impacting liver function. The liver becomes nodular, and its ability to perform vital tasks such as detoxifying and synthesizing essential proteins is compromised [14].

Impact on Liver Function: The Silent Consequences

The progression of liver diseases brings about a cascade of consequences, each stage chipping away at the liver's ability to maintain homeostasis within the body. As inflammation and fibrosis set in, the liver's ability to detoxify the blood diminishes. This can result in the accumulation of toxins in the body, contributing to a range of complications, including hepatic encephalopathy and impaired cognitive function [14]. Cirrhosis disrupts the liver's capacity to synthesize proteins essential for blood clotting and maintaining nutritional balance. This can lead to a higher risk of bleeding disorders and malnutrition, further complicating the overall health of individuals with advanced liver diseases [15]. Cirrhosis induces changes in blood flow within the liver, leading to portal hypertension. Increased pressure in the portal vein can result in the development of varices and swollen blood vessels prone to bleeding. This complication adds another layer of complexity to advanced liver diseases [15].

Hepatocellular Carcinoma Risk

One of the gravest risks associated with advanced liver disease stages is the development of HCC, a primary liver cancer. The risk of HCC significantly escalates in the presence of cirrhosis or advanced fibrosis. Cirrhosis acts as a breeding ground for HCC, with up to 80-90% of HCC cases occurring in individuals with underlying cirrhosis [16]. The mechanisms linking cirrhosis to HCC are multifaceted, involving the cumulative impact of chronic inflammation, genetic factors, and alterations in the liver microenvironment [16]. While cirrhosis is a primary risk factor, other factors contribute to HCC development. Chronic infection with hepatitis B or C viruses, excessive alcohol consumption, and NAFLD are additional risk factors that can independently or synergistically elevate the likelihood of HCC [16,17].

Strategies for Early Detection: Navigating the Challenges

Given the grave prognosis associated with advanced liver diseases and HCC, early detection becomes paramount. Several strategies aim to identify these conditions at a stage where interventions can be more effective. Surveillance programs involve regular screening of individuals at high risk for liver diseases, particularly those with cirrhosis or chronic viral hepatitis. Imaging studies, such as ultrasounds, magnetic resonance imaging (MRI), and blood tests assessing biomarkers, play a crucial role in detecting early signs of HCC [17]. Technological advancements in imaging, such as contrast-enhanced ultrasound and computed tomography (CT) scans, contribute to more precise and early detection of liver lesions. These modalities enable healthcare professionals to identify abnormalities at a smaller size, enhancing the chances of successful intervention [17]. Ongoing research focuses on identifying biomarkers and genetic profiles associated with an increased risk of HCC. Integrating these markers into routine screening protocols can enhance the accuracy of early detection and tailor interventions to individualized risks [18]. By understanding the stages of liver disease progression, appreciating the impact on liver function, and recognizing the looming threat of HCC, healthcare professionals and individuals alike can collaborate to implement effective surveillance strategies. Through continued research, technological advancements, and a commitment to holistic patient care, we pave the way for a future where liver diseases are detected early, managed effectively, and, ultimately, prevented.

Current diagnostic trends

This journey encompasses the pivotal role of advanced imaging techniques in accurate diagnosis and staging, the exciting landscape of emerging innovations in liver imaging, and the intricate world of serological biomarkers (the current markers for liver diseases with their limitations and the ongoing quest for advancements). The realm of liver diagnostics has witnessed a revolution with the advent of advanced imaging techniques. These modalities play a crucial role in accurately diagnosing liver conditions and staging the severity of diseases. Let us navigate through the key players in this imaging symphony. Advanced imaging techniques, such as CT, MRI, and elastography, have redefined our ability to visualize the liver in unprecedented detail. These techniques provide a window into the organ's internal structure, helping clinicians identify abnormalities, assess the extent of liver damage, and formulate precise treatment plans. CT imaging offers a comprehensive view of the liver, allowing for the identification of lesions, vascular abnormalities, and signs of cirrhosis. Using contrast agents enhances the visibility of blood vessels and aids in distinguishing between different liver tissues [19]. MRI, with its superior soft tissue contrast, enables a detailed assessment of liver morphology. It is precious in distinguishing between different liver lesions, characterizing tissue types, and detecting early signs of liver diseases [19]. Elastography measures the stiffness of liver tissue, providing insights into the degree of fibrosis. This non-invasive method has become integral in staging liver diseases, especially in conditions such as cirrhosis, where the liver's elasticity is compromised [19].

Emerging Innovations in Liver Imaging: Paving the Way for Precision

The landscape of liver imaging continues to evolve, with innovations promising even greater precision and diagnostic capabilities. Emerging technologies offer a glimpse into the future of liver diagnostics. Three-dimensional (3D) imaging techniques bring a new dimension to liver diagnostics, offering enhanced spatial understanding. This innovation holds promise in surgical planning, allowing for more accurate interventions and improved outcomes [20]. Functional imaging techniques focus on assessing liver function beyond mere anatomy. Approaches such as diffusion-weighted imaging (DWI) provide information about tissue cellularity and microstructure, contributing to a more comprehensive evaluation of liver health [20]. Integrating AI in liver imaging is a game-changer. AI algorithms can analyze vast imaging data, thus aiding in early disease detection, precise staging, and personalized treatment planning [20].

Serological Biomarkers: Decoding the Molecular Language of Liver Diseases

While imaging techniques offer a window into the structural aspects of the liver, serological biomarkers delve into the molecular language of liver diseases. These blood-based markers provide valuable information about the liver's function, inflammation, and the risk of underlying conditions. Serological biomarkers encompass diverse molecules reflecting different aspects of liver health. Typical markers include liver enzymes, proteins, and genetic factors that, when measured in blood, offer valuable insights into the ongoing status of the liver. Aspartate aminotransferase (AST) and alanine aminotransferase (ALT) are classic markers of liver function. Elevated levels indicate liver inflammation or injury. However, these enzymes are not specific to liver diseases, and other factors, such as muscle damage, can also influence their levels [21]. Bilirubin, a yellow pigment, is a marker of liver excretory function. Elevated levels can lead to jaundice, indicating impaired bilirubin metabolism and potential liver dysfunction [21]. Alpha feto-protein (AFP) is a tumor marker associated with HCC. While elevated levels can indicate the presence of HCC, AFP has limitations, and additional imaging studies are often required for a definitive diagnosis [21].

Limitations and Ongoing Research: The Quest for Precision

Despite their utility, serological biomarkers have inherent limitations. Their lack of specificity, sensitivity, and inability to provide real-time insights necessitate ongoing research to refine existing markers and discover novel ones. Liver enzymes such as AST and ALT lack specificity to the liver, as their elevation can result from various conditions. This poses challenges in pinpointing the exact cause of liver abnormalities based solely on enzyme levels [21]. While a valuable marker for HCC, AFP has limitations, including its elevation in conditions other than HCC and instances of HCC without AFP elevation. Ongoing research explores the integration of AFP with other biomarkers for enhanced diagnostic accuracy [22]. Ongoing research aims to discover novel biomarkers that offer enhanced specificity and sensitivity. The advent of omics technologies, including genomics, proteomics, and metabolomics, holds promise in identifying molecular signatures that accurately reflect liver health. As advanced imaging techniques evolve, providing unprecedented clarity and precision, and as serological biomarkers undergo refinement and expansion, the landscape of liver diagnostics holds promise for more accurate, timely, and personalized approaches to patient care. The journey into the intricacies of liver health is marked by continuous innovation, which is fueled by the collective quest for better diagnostic tools and a deeper understanding of the molecular intricacies within our resilient organ [22].

Molecular diagnostics

In this exploration, we delve into genetic markers, their potential role in personalized medicine, and their profound implications for treatment decisions. Through the lens of molecular diagnostics, we decipher the genetic tapestry that influences our liver's journey toward health or disease.

Genetic Markers: Unraveling the Blueprint of Liver Health

The human genome, a remarkable blueprint encoded in our DNA, serves as the master script guiding the orchestration of life. In the context of liver health, genetic markers provide a unique window into the interplay of our genetic makeup with the complex factors influencing liver function. Our genetic code, comprised of DNA sequences, holds the instructions for building and maintaining every aspect of our bodies, including the liver. Genetic markers, specific variations in our DNA, act as signposts, revealing potential susceptibilities, risks, and protective factors concerning liver health [23]. The advent of molecular diagnostics has ushered in an era of personalized medicine, where treatments are tailored to an individual's unique genetic makeup. In liver health, understanding genetic markers enables healthcare providers to customize diagnostic approaches, predict disease trajectories, and optimize treatment strategies for each patient [23].

Genetic Variations and Disease Risk

Certain genetic variations can predispose individuals to liver diseases. For instance, polymorphisms in genes associated with alcohol metabolism may influence an individual's susceptibility to ALD [24]. By identifying these variations, clinicians can assess disease risk and implement preventive measures. Genetic markers are pivotal in pharmacogenomics, where drug therapies are tailored based on an individual's genetic profile. In liver diseases, understanding how a patient's genes influence drug metabolism allows for the selection of medications with optimal efficacy and minimal side effects [24]. The insights from genetic markers extend beyond risk assessment, shaping the decisions that guide the treatment journey. By unraveling the genetic landscape, clinicians can make informed choices that optimize therapeutic outcomes and minimize adverse effects. Molecular diagnostics empowers healthcare providers to adopt precise therapeutic approaches by targeting the genetic culprits driving liver diseases. In conditions such as hereditary hemochromatosis, where genetic mutations cause iron overload, early detection allows for interventions to prevent complications such as cirrhosis [25].

Predictive Genetic Testing: Anticipating Disease Trajectories

Predictive genetic testing provides a glimpse into the future, anticipating disease trajectories before symptoms manifest. In the context of liver diseases, this is particularly relevant for conditions with a genetic component, such as Wilson's disease. Identifying genetic markers early on allows for proactive management, potentially altering the course of the disease [25]. Understanding the genetic underpinnings of liver diseases goes beyond pharmacological interventions. It extends to lifestyle modifications, where individuals can receive personalized guidance based on their genetic predispositions. For instance, individuals with a genetic predisposition to NAFLD may benefit from tailored dietary recommendations and exercise regimens [24,25]. While the promises of molecular diagnostics are vast, navigating the genetic landscape comes with challenges and ethical considerations. As we peer into the genetic tapestry, we must tread carefully to ensure the responsible and equitable application of genetic insights. The integration of genetic information into healthcare requires a supportive framework, and genetic counseling emerges as a vital component. Genetic counselors guide individuals and families, addressing the psychological, ethical, and social implications of genetic testing results. The power to unveil genetic information demands a delicate balance between scientific discovery and moral responsibility. Privacy, informed consent, and the potential for genetic discrimination underscore the need for robust ethical frameworks to safeguard individuals as they navigate the genetic landscape [25]. The sensitive nature of genetic information raises concerns about privacy and the need for informed consent. Individuals undergoing genetic testing must be fully aware of the potential implications of the results and have agency in deciding how their genetic information is used. The fear of genetic discrimination, where individuals may face prejudice based on genetic information, necessitates legal protection. Ensuring that genetic data does not result in discriminatory practices is crucial for fostering trust in applying molecular diagnostics [26].

Contemporary therapeutic approaches

This section encompasses the diverse strategies employed in the quest for optimal liver function, addressing challenges, embracing opportunities, and recognizing the crucial role of patients in shaping their well-being. Our exploration will traverse pharmacological interventions, lifestyle modifications, and surgical interventions, each playing a unique part in the intricate dance of liver health.

Pharmacological Interventions: Medications Paving the Path to Liver Wellness

Contemporary pharmacological interventions form the backbone of liver disease management. From antiviral agents for viral hepatitis to medications alleviating symptoms of cirrhosis, the pharmacological arsenal is vast. For viral hepatitis, antiviral medications are pivotal. Drugs such as tenofovir and entecavir have revolutionized the treatment landscape for chronic hepatitis B, suppressing viral replication and reducing the risk of complications [26]. The advent of DAAs has transformed the treatment of hepatitis C. Drugs such as sofosbuvir and ledipasvir offer high cure rates with minimal side effects, marking a significant leap forward in the management of this once-challenging condition. Autoimmune liver diseases, such as autoimmune hepatitis, often necessitate immunosuppressive medications such as prednisone or azathioprine. These drugs modulate the immune response, mitigating inflammation and preventing further damage [26]. Despite the strides made in pharmacological interventions, challenges persist. Balancing the efficacy of medications with potential adverse effects remains a delicate task. The emergence of antiviral resistance poses a challenge in the long-term management of viral hepatitis. Continuous research is vital to developing new antiviral agents to overcome resistance and suppress sustained viral suppression [26]. Immunosuppressants, while effective, come with potential side effects, including an increased risk of infections and metabolic disturbances. Careful monitoring and personalized treatment plans are essential to balance therapeutic efficacy and minimize adverse effects. The cost and accessibility of medications pose significant challenges, especially in resource-limited settings. Efforts to bridge these gaps involve advocating for equitable access to essential medicines and exploring innovative pricing models [25,26].

Lifestyle Modifications: The Power of Personal Choices in Liver Wellness

Lifestyle modifications, particularly dietary changes, are pivotal in supporting liver health. A well-balanced diet can mitigate the impact of liver diseases and promote overall well-being. For individuals with liver diseases, especially those with ALD, reducing or eliminating alcohol consumption is paramount. Alcohol places an additional burden on an already compromised liver and can exacerbate inflammation and scarring [27]. A diet rich in fruits, vegetables, whole grains, and lean proteins provides essential nutrients that support liver function. Nutritional interventions are particularly relevant in conditions such as NAFLD, where dietary choices significantly impact disease progression [27]. Physical activity emerges as a potent ally in the quest for liver wellness. Regular exercise not only contributes to overall health but also exerts specific benefits on liver function. Obesity is a significant risk factor for NAFLD. Engaging in regular physical activity contributes to weight management, reducing the risk of NAFLD and its progression to more severe forms, such as NASH [27]. Physical activity enhances insulin sensitivity, a crucial factor in managing liver diseases. Improved insulin sensitivity reduces the risk of metabolic complications associated with liver conditions such as cirrhosis. Beyond specific liver-related benefits, physical activity contributes to enhanced overall well-being. It improves cardiovascular health, boosts mental health, and fosters a sense of vitality, positively influencing the holistic picture of health [27].

Surgical Interventions: Navigating Advanced Therapeutic Avenues

In cases where liver diseases progress to an advanced stage and no other therapeutic options remain, liver transplantation emerges as a lifesaving intervention. Liver transplantation is indicated for conditions such as end-stage liver disease, acute liver failure, or certain liver cancers. Rigorous evaluation and adherence to specific criteria ensure that liver transplantation is reserved for those most benefit [28]. Despite the success of liver transplantation, challenges persist, primarily in the availability of donor organs. Efforts to increase organ donation rates, explore living donor options, and advance research in artificial and bioengineered livers are underway to address this critical gap. Beyond transplantation, various surgical options exist to address specific liver conditions, offering targeted interventions for improved outcomes. In cases of liver tumors, surgical resection involves removing the affected portion of the liver. This approach is particularly relevant in HCC, providing a curative option if the tumor is localized and the remaining liver function is sufficient [28]. Conditions such as primary biliary cirrhosis may lead to bile duct obstruction. Biliary surgery, including procedures to address strictures or obstructions, aims to restore proper bile flow and alleviate symptoms.

Patient Selection Criteria: Shaping Therapeutic Paths

Whether considering pharmacological interventions, lifestyle modifications, or surgical interventions, patient selection is critical in shaping therapeutic paths. A multidisciplinary approach involving hepatologists, surgeons, nutritionists, and other specialists ensures comprehensive evaluation. This collaborative effort allows for a holistic understanding of the patient's condition and facilitates informed, patient-centered decision-making [29]. Stratifying patient risks involves assessing the severity of liver disease, evaluating comorbidities, and considering individual factors such as age and lifestyle. This personalized approach allows for tailoring interventions to each patient's unique needs. Informed consent is a cornerstone of ethical medical practice. Patients should be provided with clear, understandable information about potential risks, benefits, and alternatives to empower them in making informed decisions regarding their therapeutic journey [29].

Personalized medicine in liver disease

This revolutionary approach to healthcare seeks to tailor treatments to individual profiles, acknowledging the unique genetic, molecular, and lifestyle factors that shape each person's health journey. In this chapter, we delve into the importance of precision medicine, explore case studies, and celebrate success stories illuminating the power of personalized interventions in liver disease.

Importance of Precision Medicine: Navigating the Complexities of Liver Health

Precision medicine in liver disease begins with a profound understanding of the genetic mosaic that makes each unique. Our genetic makeup influences our susceptibility to liver diseases and our response to treatments. Unraveling this genetic code empowers healthcare providers to tailor interventions with unparalleled precision [29]. Genetic variations can significantly impact how individuals respond to medications. For instance, specific polymorphisms may influence the metabolism of drugs used in liver disease treatment, guiding clinicians in selecting the most effective and well-tolerated therapies for each patient [28,29]. Predictive genetic markers play a crucial role in anticipating treatment outcomes. Identifying characteristics that predict response to antiviral medicines in hepatitis or immunomodulatory treatments in autoimmune liver diseases allows for a personalized approach that maximizes efficacy and minimizes side effects [29]. Omics technologies, encompassing genomics, proteomics, and metabolomics, provide a comprehensive view of the molecular landscape within our bodies. By decoding molecular signatures, these technologies offer insights into the intricate mechanisms underlying liver diseases, paving the way for targeted and personalized interventions [29].

Genomics: Mapping the Genetic Blueprint

Genomic studies contribute to mapping the genetic blueprint of liver diseases. Whole-genome sequencing and genome-wide association studies unravel genetic factors influencing disease susceptibility and treatment responses, guiding the development of precision therapies [28]. Proteomics and metabolomics delve into the functional aspects of liver diseases. By studying proteins and metabolites in the liver, researchers gain insights into disease mechanisms and identify potential targets for personalized interventions [29].

Case Studies and Success Stories: Illuminating the Power of Personalized Interventions

In chronic hepatitis B, personalized medicine has demonstrated its efficacy in tailoring antiviral therapies. Case studies reveal that individuals with specific genetic variations may respond differently to antiviral medications. Tailoring treatment regimens based on these genetic profiles enhances efficacy and reduces the risk of resistance. A notable success story involves the optimization of tenofovir treatment. By considering the genetic variations in drug metabolism, clinicians have successfully tailored tenofovir doses for individual patients, achieving sustained viral suppression with minimal side effects. HCC, a common form of liver cancer, presents a complex landscape for personalized interventions. Recent advancements in genomic profiling have identified specific mutations driving HCC. Personalized therapies targeting these mutations, such as tyrosine kinase inhibitors, promise to improve outcomes and extend survival in HCC patients [30]. A compelling success story revolves around the use of sorafenib in HCC. By identifying specific mutations in the tumor, clinicians can tailor sorafenib treatment to target these mutations, leading to improved response rates and prolonged survival in select patient populations [30].

Autoimmune Liver Diseases: Navigating the Landscape With Immunomodulation

Autoimmune liver diseases, such as autoimmune hepatitis and primary biliary cholangitis, highlight the potential of immunomodulation in personalized medicine. Case studies showcase how understanding individual immune responses and genetic predispositions can guide the selection of immunosuppressive therapies, achieving disease control while minimizing side effects. Personalizing immunosuppression involves tailoring the choice and dosage of medications based on individual immune profiles. This approach has led to the successful management of autoimmune liver diseases, allowing patients to achieve remission with fewer side effects and a better quality of life [30].

Emerging therapies: pioneering the future of liver health

As we move into the ninth chapter of our exploration, we stand on the cusp of innovation, where emerging therapies hold the promise of revolutionizing the landscape of liver health. Gene editing technologies and immunomodulatory strategies are at the forefront, offering unprecedented opportunities for precision interventions. However, as we embrace these advancements, ethical considerations underscore the need for responsible and equitable applications.

Gene Editing: Redefining Possibilities in Liver Disease Treatment

The advent of CRISPR-Cas9, a groundbreaking gene editing technology, opens new avenues for precision interventions in liver diseases. CRISPR-Cas9 allows scientists to modify or correct genes precisely, holding immense potential for treating genetic liver disorders, viral infections, and even certain types of liver cancers [27]. CRISPR-Cas9 offers a potential cure for chronic hepatitis B by directly targeting the viral genome. Research is underway to develop strategies that enable CRISPR-Cas9 to edit the hepatitis B virus within infected liver cells, potentially eradicating the virus and achieving a functional cure [27]. Specific genetic mutations result in inherited liver disorders, such as hemochromatosis or alpha-1 antitrypsin deficiency. Gene editing promises to correct these genetic defects at the source, offering a curative approach that addresses the root cause of the disorders [27]. As we venture into gene editing, ethical considerations become paramount. The power to manipulate the genetic code demands scrutiny to ensure responsible and equitable applications. Germline editing, which involves modifying the DNA of sperm, eggs, or embryos, raises profound ethical questions. While it holds the potential to eliminate genetic diseases from future generations, concerns about unintended consequences and the potential for designer babies necessitate careful moral deliberation [28]. The accessibility of gene editing therapies is a critical ethical consideration. Ensuring equitable access to these revolutionary treatments is essential to prevent the exacerbation of existing healthcare disparities and to promote global health equity [28].

Immunomodulation: Harnessing the Immune System for Liver Health

Immunomodulation, a form of immunotherapy, represents a paradigm shift in the approach to liver diseases. Immunomodulatory strategies aim to enhance anti-tumor responses, control autoimmune reactions, and combat viral infections with remarkable specificity by harnessing the body's immune system. Checkpoint inhibitors, a type of immunomodulatory drug, have shown promise in liver cancers, including HCC. By blocking certain immune checkpoints, these drugs unleash the full potential of the immune system to target and destroy cancer cells. Autoimmune liver diseases arise from an overactive immune response against the liver [29]. Immunomodulatory therapies, such as corticosteroids or newer biologic agents, restore immune balance, mitigating inflammation and preventing further damage to the liver. The landscape of immunomodulation is dynamic, with ongoing clinical trials exploring novel therapies and expanding the applications of existing ones. Ongoing research focuses on combining different immunomodulatory agents to create synergistic effects. By leveraging the strengths of multiple therapies, clinicians aim to enhance treatment efficacy and overcome resistance, especially in conditions such as advanced liver cancers. The future of immunomodulation in liver diseases lies in personalized approaches. Researchers are exploring ways to identify specific immune profiles that predict responses to immunotherapy, allowing for tailoring treatments to maximize individual patient benefits [29].

Future directions

The landscape of liver health is evolving, and the future promises groundbreaking advancements that will redefine how we approach diagnostics and patient care. In exploring future directions, we delve into the realms of novel biomarkers and diagnostics, the integration of telemedicine and digital health, and the potential of remote monitoring and management. Collectively, these elements represent the innovative path forward for liver health, offering hope for improved detection, personalized care, and enhanced patient outcomes.

Novel Biomarkers and Diagnostics: Pioneering the Way

The need for early detection and precise monitoring of liver diseases propels the quest for novel biomarkers and diagnostics. Ongoing research is unraveling promising developments that could revolutionize identifying and understanding these conditions. Genomic and proteomic approaches are at the forefront of identifying novel biomarkers. By decoding the molecular tapestry of liver diseases, researchers aim to discover unique signatures that can serve as early indicators, allowing for timely intervention and personalized treatment [30]. Liquid biopsies, which involve analyzing circulating biomarkers in bodily fluids, offer a non-invasive window into liver health. This approach could replace or complement traditional liver biopsies, providing valuable insights into disease progression and treatment response. MicroRNAs, which regulate gene expression, are emerging as potential biomarkers. Altered microRNA signatures have been linked to various liver diseases, offering a glimpse into their diagnostic potential and the intricate molecular changes occurring in diseased livers [30]. While the promise of novel biomarkers is exciting, translating these discoveries into clinical practice comes with challenges. Rigorous validation and standardization processes are essential to ensure the reliability of novel biomarkers. Robust studies confirming these markers' accuracy and reproducibility are crucial before they can be integrated into routine clinical practice. The ethical implications of using novel biomarkers must be carefully considered. Issues such as patient consent, data privacy, and the potential for overdiagnosis need thoughtful exploration to ensure that the integration of these tools aligns with ethical principles [30].

Telemedicine and Digital Health: Bridging Gaps for Enhanced Patient Care

Telemedicine and digital health have emerged as transformative forces in healthcare, offering opportunities to bridge gaps in access, provide remote consultations, and enhance overall patient care. Virtual consultations enable individuals to access specialized liver care regardless of their geographical location. This is particularly significant for those in remote or underserved areas, opening avenues for expert opinions and reducing disparities in healthcare access. The rise of health apps and wearables empowers patients to participate actively in their health journey. These tools can monitor vital signs, track medication adherence, and provide real-time data to healthcare providers, fostering a collaborative approach to liver health management. Digital health records facilitate seamless communication among healthcare providers. This integration enhances care coordination, ensuring that all patient healthcare team members have access to the latest information, leading to more informed decision-making. Despite the transformative potential of telemedicine and digital health, challenges persist. The digital divide, characterized by uneven access to technology, poses a challenge. Efforts are needed to ensure that all individuals, regardless of socioeconomic status, access the tools required for telemedicine and digital health participation [31]. As digital health relies on collecting and transmitting sensitive patient information, ensuring robust data security measures is paramount. Striking a balance between accessibility and safeguarding patient privacy is crucial for fostering trust in digital healthcare solutions [31].

Remote Monitoring and Management: Empowering Patient-Centric Care

Remote monitoring and management present a paradigm shift in healthcare by providing real-time insights into patients' liver health, enabling proactive interventions and personalized care plans. Remote monitoring allows for the continuous tracking of biomarkers indicative of liver health. This real-time data enables healthcare providers to detect subtle changes early, facilitating prompt treatment plans and intervention adjustments. Smart devices designed for home monitoring empower patients to participate actively in their care. From smart scales that track weight fluctuations to instruments measuring blood pressure or glucose levels, these tools offer a holistic view of a patient's health status. Integrating AI in remote management brings predictive analytics to the forefront. AI algorithms can analyze large datasets to identify patterns and predict disease progression, allowing for proactive interventions before symptoms manifest [32-36]. While remote monitoring holds immense promise, consideration must be given to balancing patient autonomy and the need for support. Remote monitoring is most effective when patients are actively engaged in their care. Empowering patients with education and involving them in decision-making ensures that remote monitoring becomes a tool for collaboration rather than a passive surveillance system. Successfully integrating remote monitoring into existing healthcare systems requires overcoming implementation hurdles. Ensuring interoperability, addressing regulatory considerations, and providing adequate training for healthcare professionals are key factors in seamlessly adopting remote management strategies [36-40].

## Conclusions

As we navigate the complexities of liver health, we uncover the current state of affairs and the bright future that awaits us. Contemporary patterns portend a paradigm shift in the field of diagnostics. Sophisticated imaging, molecular diagnostics, and serological biomarkers provide intricate understandings of liver diseases, wherein precision is paramount. Modern therapeutic approaches emphasize the significance of individualized interventions. Pharmacological interventions, lifestyle adjustments, and surgical alternatives are among the many methods utilized to address the unique requirements of each patient. Promising developments are anticipated in the field of precision medicine. Using genetic markers to direct therapies will inaugurate an age of targeted and individualized interventions. Protein modification and immunomodulation, poised to revolutionize the treatment of liver disease, emerge as beacons of hope. These innovations will be guided toward responsible applications by ethical considerations. The amalgamation of telemedicine and digital health represents a fundamental change, as it eliminates obstacles to access, grants patients greater agency, and improves care coordination. In the future, remote monitoring is anticipated to have a significant impact by providing timely insights, encouraging patient participation, and guiding the way toward proactive, patient-focused healthcare. By embracing curiosity and allocating resources towards rigorous research endeavors, we can unravel the enigmas surrounding liver health. Profound advancements in biomarkers, diagnostics, and therapies result from ceaseless investigation. By establishing cross-disciplinary, cross-institutional, and cross-border partnerships and using collaborative endeavors, we can advance the discipline and guarantee a future in which liver health is not merely monitored but genuinely fostered.
